# 2,2′-Dinitro-5,5′-dithio­dibenzoic acid

**DOI:** 10.1107/S1600536808031620

**Published:** 2008-10-04

**Authors:** Li-Jin Wang

**Affiliations:** a Department of Chemistry, Lishui University, Lishui 323000, People’s Republic of China

## Abstract

In the title compound, C_14_H_8_N_2_O_8_S_2_, the asymmetric unit contains two independent 2,2′-dinitro-5,5′-dithio­dibenzoic acid (Dina) mol­ecules with roughly the same conformation. In the crystal structure, strong inter­molecular O—H⋯O hydrogen bonds link the organic mol­ecules into a one-dimensional zigzag chain along the *a* axis. The dihedral angles between the two aromatic rings [109.3 (2) and 103.1 (3)°] are larger than that (88.95°) observed in a structure of the compound with a solvent water mol­ecule [Shefter & Kalman (1969), *J. Chem. Soc. D*, p. 1027]. Such a difference may be explained by the occurrence of O—H⋯O hydrogen bonds involving the water mol­ecule in the previously reported structure.

## Related literature

For general background, see: Gudbjarlson *et al.* (1991 ▶); Li *et al.* (2006 ▶); Luo *et al.* (2007 ▶); Ye *et al.* (2005 ▶). For a related structure, see: Shefter & Kalman (1969 ▶)
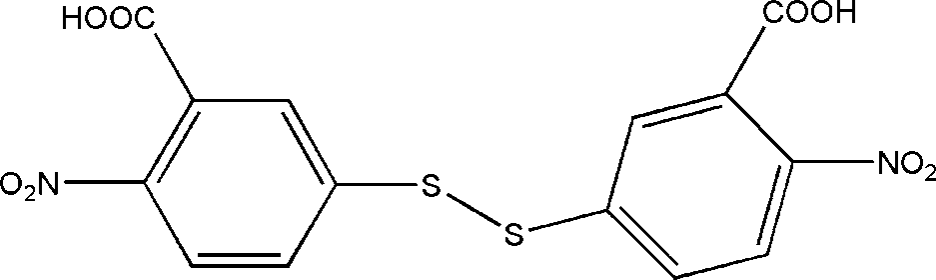


## Experimental

### 

#### Crystal data

C_14_H_8_N_2_O_8_S_2_
*M* *_r_* = 396.34
Triclinic, 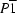

*a* = 7.875 (2) Å
*b* = 14.695 (4) Å
*c* = 15.116 (5) Å
α = 111.480 (5)°
β = 101.182 (5)°
γ = 90.572 (5)°
*V* = 1590.6 (8) Å^3^
*Z* = 4
Mo *K*α radiation
μ = 0.39 mm^−1^
*T* = 298 (2) K
0.21 × 0.17 × 0.15 mm

#### Data collection

Bruker APEXII area-detector diffractometer
Absorption correction: multi-scan (*SADABS*; Sheldrick, 1996 ▶) *T*_min_ = 0.921, *T*_max_ = 0.942
8041 measured reflections
5555 independent reflections
3077 reflections with *I* > 2σ(*I*)
*R*_int_ = 0.031

#### Refinement

*R*[*F* ^2^ > 2σ(*F* ^2^)] = 0.051
*wR*(*F* ^2^) = 0.171
*S* = 0.92
5555 reflections
473 parameters
H-atom parameters constrained
Δρ_max_ = 0.37 e Å^−3^
Δρ_min_ = −0.47 e Å^−3^

### 

Data collection: *APEX2* (Bruker, 2004 ▶); cell refinement: *APEX2*; data reduction: *SAINT* (Bruker, 2004 ▶); program(s) used to solve structure: *SHELXS97* (Sheldrick, 2008 ▶); program(s) used to refine structure: *SHELXL97* (Sheldrick, 2008 ▶); molecular graphics: *ORTEPIII* (Burnett & Johnson, 1996 ▶), *ORTEP-3 for Windows* (Farrugia, 1997 ▶) and *PLATON* (Spek, 2003 ▶); software used to prepare material for publication: *SHELXL97*.

## Supplementary Material

Crystal structure: contains datablocks I, global. DOI: 10.1107/S1600536808031620/dn2385sup1.cif

Structure factors: contains datablocks I. DOI: 10.1107/S1600536808031620/dn2385Isup2.hkl

Additional supplementary materials:  crystallographic information; 3D view; checkCIF report

## Figures and Tables

**Table 1:** Hydrogen-bond geometry (Å, °)

*D*—H⋯*A*	*D*—H	H⋯*A*	*D*⋯*A*	*D*—H⋯*A*
O11—H1*A*⋯O22^i^	0.82	1.83	2.635 (4)	166
O21—H2*A*⋯O12^ii^	0.82	1.88	2.688 (4)	169
O31—H3*A*⋯O42^i^	0.82	1.85	2.666 (4)	171
O41—H4*A*⋯O32^ii^	0.82	1.83	2.618 (4)	160

Symmetry codes: (i) 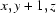; (ii) 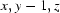.

**Fig. 1.**
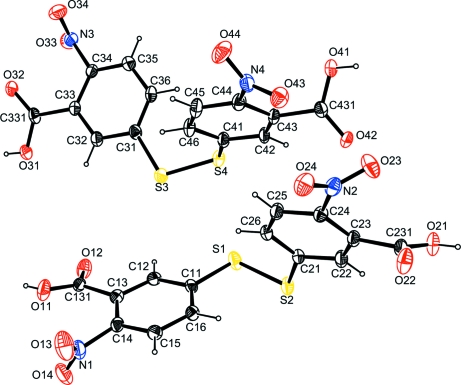
Molecular view of (I) with the atom-labeling scheme. scheme. Ellipsoids are drawn at the 30% probability level. H atoms are represented as small spheres of arbitrary radii..

**Fig. 2.**
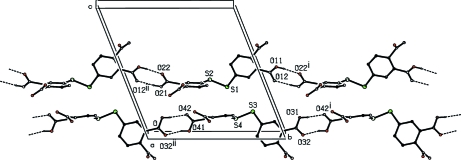
Partial packing view showing the formation of the chain though O—H···O hydrogen bonds. H bonds are shown as dashed line. H atoms not involved in H bonds have been omitted for clarity. [Symmetry codes: (i) x, y + 1, z; (ii) x, y - 1, z.]

## supplementary crystallographic information

### Comment

Until now, various flexible carboxylate ligands can often be employed due to their versatile coordination modes and high structural stability. In particular, multi-benzenecarboxylate ligands have been shown to be good building blocks in the design of metal-organic materials (Li *et al.*, 2006; Luo *et al.*, 2007; Gudbjarlson *et al.*, 1991). On the other hand, relative strong hydrogen bondings play an important role in the formation of the ultimate netwrok (Ye *et al.*, 2005). Originally, we attempted to synthesize a complex in the presence of metal salt. However, we only obtain the starting organic material (I).

In the title compound, [C_14_H_8_N_2_O_8_S]_2_, the asymmetric unit is built up by two independent 5,5'-dithiobis(2-nitrobenzcic-acid) (Dina) molecules having roughly the same conformation (Fig. 1). The geometric parameters for (I) are in the usual range. The dihedral angles between the two aromatic rings in the molecules are 109.3 (2)° and 103.1 (3)°, respectively, which indicate a twisted conformation between phenyl rings. A similar [C_14_H_8_N_2_O_8_S, H_2_O] compound, has been previously published (Shefter & Kalman, 1969). In this previous structure, the occurrence of the water molecule which acts as donor and acceptor, links the organic molecules to build up a sheet whereas in the title compound, the O—H···O hydrogen bonds form chains which develop parallel to the *a* axis (Table 1, Fig.2). In the previously reported compound, [C_14_H_8_N_2_O_8_S, H_2_O], the dihedral angles between the two aromatic rings is slightly smaller (88.95°) than in the title compound. Such difference might result from the occurrence of the water molecule which interconnects the oragnic molecule.

### Experimental

5,5'-dithiobis(2-nitrobenzcic-acid) (21 mg,0.05 mmol), CoSo4 (13 mg, 0.06 mmol). were added in methanol. The mixture was refluxed under stirring for six hours. After cooling the resulting mixture to room temperature, some single crystals appeared within two weeks.

### Refinement

All H atoms attached to C and O atoms were fixed geometrically and treated as riding with C—H = 0.93 Å (aromatic) and O—H = 0.82 Å with *U*_iso_(H) = 1.2*U*_eq_(C, O).

### Crystal data

**Table:**

C_14_H_8_N_2_O_8_S_2_	*Z* = 4
*M**_r_* = 396.34	*F*(000) = 808
Triclinic, *P*1	*D*_x_ = 1.655 Mg m^−^^3^
Hall symbol: -P 1	Mo *K*α radiation, λ = 0.71073 Å
*a* = 7.875 (2) Å	Cell parameters from 5555 reflections
*b* = 14.695 (4) Å	θ = 1.5–25.1°
*c* = 15.116 (5) Å	µ = 0.39 mm^−^^1^
α = 111.480 (5)°	*T* = 298 K
β = 101.182 (5)°	Block, colourless
γ = 90.572 (5)°	0.21 × 0.17 × 0.15 mm
*V* = 1590.6 (8) Å^3^	

### Data collection

**Table:**

Bruker APEXII area-detector diffractometer	5555 independent reflections
Radiation source: fine-focus sealed tube	3077 reflections with *I* > 2σ(*I*)
graphite	*R*_int_ = 0.031
φ and ω scans	θ_max_ = 25.1°, θ_min_ = 1.5°
Absorption correction: multi-scan (SADABS; Sheldrick, 1996)	*h* = −9→9
*T*_min_ = 0.921, *T*_max_ = 0.942	*k* = −17→16
8041 measured reflections	*l* = −18→15

### Refinement

**Table:**

Refinement on *F*^2^	Primary atom site location: structure-invariant direct methods
Least-squares matrix: full	Secondary atom site location: difference Fourier map
*R*[*F*^2^ > 2σ(*F*^2^)] = 0.051	Hydrogen site location: inferred from neighbouring sites
*wR*(*F*^2^) = 0.171	H-atom parameters constrained
*S* = 0.92	*w* = 1/[σ^2^(*F*_o_^2^) + (0.1043*P*)^2^] where *P* = (*F*_o_^2^ + 2*F*_c_^2^)/3
5555 reflections	(Δ/σ)_max_ < 0.001
473 parameters	Δρ_max_ = 0.37 e Å^−^^3^
0 restraints	Δρ_min_ = −0.46 e Å^−^^3^

### Special details

**Table:**

Geometry. All e.s.d.'s (except the e.s.d. in the dihedral angle between two l.s. planes) are estimated using the full covariance matrix. The cell e.s.d.'s are taken into account individually in the estimation of e.s.d.'s in distances, angles and torsion angles; correlations between e.s.d.'s in cell parameters are only used when they are defined by crystal symmetry. An approximate (isotropic) treatment of cell e.s.d.'s is used for estimating e.s.d.'s involving l.s. planes.
Refinement. Refinement of *F*^2^ against ALL reflections. The weighted *R*-factor *wR* and goodness of fit *S* are based on *F*^2^, conventional *R*-factors *R* are based on *F*, with *F* set to zero for negative *F*^2^. The threshold expression of *F*^2^ > σ(*F*^2^) is used only for calculating *R*-factors(gt) *etc*. and is not relevant to the choice of reflections for refinement. *R*-factors based on *F*^2^ are statistically about twice as large as those based on *F*, and *R*- factors based on ALL data will be even larger.

### Fractional atomic coordinates and isotropic or equivalent isotropic displacement parameters (Å^2^)

**Table:**

	*x*	*y*	*z*	*U*_iso_*/*U*_eq_	
S1	−0.01726 (16)	0.70300 (8)	0.33908 (9)	0.0468 (4)	
S2	−0.04932 (15)	0.59689 (8)	0.39126 (9)	0.0433 (3)	
N1	0.3900 (5)	1.0562 (3)	0.6593 (3)	0.0487 (11)	
N2	0.5648 (5)	0.3610 (3)	0.3567 (3)	0.0430 (10)	
O11	0.3554 (4)	1.1261 (2)	0.5124 (3)	0.0592 (10)	
H1A	0.3428	1.1749	0.4984	0.089*	
O12	0.1174 (4)	1.0668 (2)	0.3939 (3)	0.0524 (9)	
O13	0.5428 (5)	1.0529 (3)	0.6900 (3)	0.0876 (15)	
O14	0.3084 (5)	1.1242 (3)	0.6944 (3)	0.0700 (12)	
O21	0.1194 (4)	0.2278 (2)	0.3491 (3)	0.0538 (10)	
H2A	0.1308	0.1771	0.3597	0.081*	
O22	0.3681 (4)	0.2775 (2)	0.4591 (3)	0.0584 (10)	
O23	0.5385 (5)	0.2744 (2)	0.3064 (3)	0.0657 (11)	
O24	0.7108 (4)	0.4035 (2)	0.3973 (3)	0.0579 (10)	
C11	0.1118 (5)	0.8035 (3)	0.4363 (3)	0.0316 (10)	
C12	0.1199 (5)	0.8905 (3)	0.4200 (3)	0.0332 (10)	
H12	0.0591	0.8927	0.3617	0.040*	
C13	0.2177 (5)	0.9741 (3)	0.4898 (3)	0.0326 (10)	
C14	0.2998 (5)	0.9692 (3)	0.5775 (3)	0.0332 (10)	
C15	0.2940 (5)	0.8827 (3)	0.5935 (3)	0.0364 (11)	
H15	0.3536	0.8807	0.6521	0.044*	
C16	0.2006 (5)	0.7996 (3)	0.5234 (3)	0.0330 (10)	
H16	0.1969	0.7413	0.5342	0.040*	
C21	0.1340 (5)	0.5290 (3)	0.3762 (3)	0.0340 (11)	
C22	0.1208 (6)	0.4352 (3)	0.3793 (3)	0.0403 (12)	
H22	0.0154	0.4094	0.3831	0.048*	
C23	0.2633 (5)	0.3803 (3)	0.3768 (3)	0.0339 (11)	
C24	0.4171 (5)	0.4200 (3)	0.3685 (3)	0.0345 (11)	
C25	0.4336 (6)	0.5117 (3)	0.3644 (3)	0.0380 (11)	
H25	0.5388	0.5362	0.3589	0.046*	
C26	0.2938 (5)	0.5661 (3)	0.3684 (3)	0.0359 (11)	
H26	0.3041	0.6282	0.3660	0.043*	
C131	0.2281 (5)	1.0620 (3)	0.4637 (3)	0.0342 (10)	
C231	0.2520 (5)	0.2874 (3)	0.3953 (3)	0.0377 (11)	
S3	0.08887 (17)	0.82074 (8)	0.15577 (9)	0.0454 (4)	
S4	0.02988 (15)	0.67435 (8)	0.09932 (9)	0.0406 (3)	
N3	0.2790 (5)	0.9447 (3)	−0.1503 (3)	0.0395 (9)	
N4	0.6705 (5)	0.4718 (3)	0.1507 (3)	0.0427 (10)	
O31	0.2018 (4)	1.1464 (2)	0.1163 (2)	0.0482 (9)	
H3A	0.2036	1.2030	0.1186	0.072*	
O32	0.3339 (4)	1.1224 (2)	−0.0084 (2)	0.0509 (9)	
O33	0.1633 (5)	0.9729 (3)	−0.1961 (3)	0.0640 (10)	
O34	0.4258 (4)	0.9351 (3)	−0.1681 (2)	0.0581 (10)	
O41	0.3935 (5)	0.3091 (2)	0.0261 (3)	0.0535 (9)	
H4A	0.3956	0.2535	0.0265	0.080*	
O42	0.2379 (4)	0.3353 (2)	0.1412 (2)	0.0441 (8)	
O43	0.6680 (4)	0.4104 (2)	0.1883 (3)	0.0530 (9)	
O44	0.7995 (4)	0.4923 (3)	0.1241 (3)	0.0688 (11)	
C31	0.1522 (6)	0.8507 (3)	0.0627 (3)	0.0363 (11)	
C32	0.1794 (5)	0.9510 (3)	0.0841 (3)	0.0377 (11)	
H32	0.1671	0.9959	0.1441	0.045*	
C33	0.2250 (5)	0.9841 (3)	0.0158 (3)	0.0330 (10)	
C34	0.2427 (5)	0.9141 (3)	−0.0736 (3)	0.0331 (10)	
C35	0.2196 (6)	0.8161 (3)	−0.0944 (4)	0.0430 (12)	
H35	0.2338	0.7712	−0.1540	0.052*	
C36	0.1747 (6)	0.7836 (3)	−0.0259 (3)	0.0412 (12)	
H36	0.1596	0.7166	−0.0394	0.049*	
C41	0.2267 (6)	0.6214 (3)	0.1214 (3)	0.0360 (11)	
C42	0.2104 (6)	0.5236 (3)	0.1103 (3)	0.0355 (11)	
H42	0.1002	0.4925	0.0981	0.043*	
C43	0.3533 (5)	0.4716 (3)	0.1167 (3)	0.0314 (10)	
C44	0.5177 (6)	0.5213 (3)	0.1368 (3)	0.0389 (11)	
C45	0.5381 (6)	0.6202 (3)	0.1518 (3)	0.0457 (13)	
H45	0.6484	0.6521	0.1665	0.055*	
C46	0.3911 (6)	0.6708 (3)	0.1446 (3)	0.0422 (12)	
H46	0.4023	0.7372	0.1552	0.051*	
C331	0.2575 (6)	1.0908 (3)	0.0403 (4)	0.0390 (12)	
C431	0.3248 (6)	0.3645 (3)	0.0970 (3)	0.0354 (11)	

### Atomic displacement parameters (Å^2^)

**Table:**

	*U*^11^	*U*^22^	*U*^33^	*U*^12^	*U*^13^	*U*^23^
S1	0.0637 (8)	0.0279 (6)	0.0414 (7)	−0.0014 (6)	−0.0062 (6)	0.0134 (6)
S2	0.0457 (7)	0.0277 (6)	0.0567 (8)	0.0010 (5)	0.0101 (6)	0.0167 (6)
N1	0.054 (3)	0.035 (2)	0.050 (3)	−0.009 (2)	0.002 (2)	0.013 (2)
N2	0.047 (2)	0.044 (3)	0.045 (2)	0.002 (2)	0.007 (2)	0.026 (2)
O11	0.067 (2)	0.045 (2)	0.071 (2)	−0.0140 (18)	0.0039 (19)	0.0339 (19)
O12	0.053 (2)	0.044 (2)	0.069 (2)	0.0025 (16)	0.0034 (18)	0.0375 (19)
O13	0.060 (3)	0.074 (3)	0.100 (3)	−0.018 (2)	−0.026 (2)	0.021 (3)
O14	0.095 (3)	0.037 (2)	0.058 (3)	−0.003 (2)	0.005 (2)	0.001 (2)
O21	0.058 (2)	0.0334 (19)	0.071 (2)	−0.0089 (16)	0.0007 (18)	0.0273 (18)
O22	0.057 (2)	0.051 (2)	0.072 (2)	−0.0115 (17)	−0.0105 (19)	0.040 (2)
O23	0.073 (2)	0.033 (2)	0.080 (3)	0.0124 (18)	0.014 (2)	0.009 (2)
O24	0.0402 (19)	0.066 (2)	0.074 (3)	0.0021 (17)	0.0096 (18)	0.035 (2)
C11	0.040 (2)	0.022 (2)	0.033 (2)	0.0046 (19)	0.009 (2)	0.0091 (19)
C12	0.037 (2)	0.032 (2)	0.035 (2)	0.0096 (19)	0.012 (2)	0.016 (2)
C13	0.031 (2)	0.032 (2)	0.038 (3)	0.0060 (19)	0.010 (2)	0.016 (2)
C14	0.027 (2)	0.030 (2)	0.043 (3)	0.0017 (18)	0.012 (2)	0.012 (2)
C15	0.044 (3)	0.033 (3)	0.034 (3)	0.007 (2)	0.008 (2)	0.016 (2)
C16	0.037 (2)	0.025 (2)	0.041 (3)	0.0098 (18)	0.011 (2)	0.015 (2)
C21	0.041 (2)	0.025 (2)	0.035 (3)	−0.0025 (19)	0.009 (2)	0.009 (2)
C22	0.051 (3)	0.028 (2)	0.039 (3)	−0.006 (2)	0.003 (2)	0.014 (2)
C23	0.041 (2)	0.026 (2)	0.035 (3)	−0.001 (2)	0.005 (2)	0.015 (2)
C24	0.037 (2)	0.032 (2)	0.038 (3)	0.003 (2)	0.008 (2)	0.017 (2)
C25	0.045 (3)	0.032 (3)	0.038 (3)	−0.005 (2)	0.007 (2)	0.016 (2)
C26	0.047 (3)	0.021 (2)	0.040 (3)	0.002 (2)	0.014 (2)	0.010 (2)
C131	0.037 (2)	0.028 (2)	0.042 (3)	0.003 (2)	0.018 (2)	0.013 (2)
C231	0.040 (2)	0.028 (2)	0.047 (3)	−0.002 (2)	0.013 (2)	0.016 (2)
S3	0.0688 (8)	0.0302 (6)	0.0422 (7)	0.0033 (6)	0.0186 (6)	0.0161 (6)
S4	0.0442 (7)	0.0307 (6)	0.0500 (7)	−0.0005 (5)	0.0062 (6)	0.0207 (6)
N3	0.046 (2)	0.038 (2)	0.037 (2)	0.0009 (19)	0.010 (2)	0.0160 (19)
N4	0.043 (2)	0.032 (2)	0.048 (2)	−0.0015 (19)	0.008 (2)	0.011 (2)
O31	0.063 (2)	0.0243 (17)	0.057 (2)	0.0022 (16)	0.0247 (18)	0.0081 (17)
O32	0.078 (2)	0.0269 (17)	0.053 (2)	−0.0034 (16)	0.0255 (19)	0.0158 (16)
O33	0.068 (2)	0.076 (3)	0.064 (2)	0.019 (2)	0.014 (2)	0.044 (2)
O34	0.058 (2)	0.072 (3)	0.051 (2)	0.0025 (19)	0.0209 (18)	0.026 (2)
O41	0.080 (2)	0.0263 (18)	0.061 (2)	−0.0009 (18)	0.030 (2)	0.0173 (18)
O42	0.0558 (19)	0.0312 (17)	0.055 (2)	−0.0011 (15)	0.0257 (17)	0.0209 (16)
O43	0.054 (2)	0.048 (2)	0.067 (2)	0.0120 (16)	0.0143 (18)	0.0325 (19)
O44	0.043 (2)	0.074 (3)	0.109 (3)	0.0074 (19)	0.035 (2)	0.047 (2)
C31	0.047 (3)	0.027 (2)	0.035 (3)	0.003 (2)	0.004 (2)	0.015 (2)
C32	0.042 (2)	0.029 (2)	0.040 (3)	−0.002 (2)	0.006 (2)	0.012 (2)
C33	0.031 (2)	0.027 (2)	0.040 (3)	−0.0004 (18)	0.005 (2)	0.013 (2)
C34	0.033 (2)	0.031 (2)	0.038 (3)	−0.0006 (19)	0.009 (2)	0.015 (2)
C35	0.051 (3)	0.034 (3)	0.043 (3)	0.001 (2)	0.013 (2)	0.012 (2)
C36	0.049 (3)	0.027 (2)	0.051 (3)	−0.001 (2)	0.010 (2)	0.019 (2)
C41	0.048 (3)	0.027 (2)	0.032 (2)	−0.005 (2)	0.003 (2)	0.014 (2)
C42	0.046 (3)	0.023 (2)	0.036 (3)	−0.007 (2)	0.004 (2)	0.012 (2)
C43	0.036 (2)	0.024 (2)	0.035 (2)	−0.0032 (19)	0.0035 (19)	0.015 (2)
C44	0.044 (3)	0.035 (3)	0.039 (3)	−0.002 (2)	0.003 (2)	0.019 (2)
C45	0.049 (3)	0.038 (3)	0.049 (3)	−0.014 (2)	−0.001 (2)	0.022 (2)
C46	0.047 (3)	0.027 (2)	0.054 (3)	−0.004 (2)	0.004 (2)	0.021 (2)
C331	0.048 (3)	0.025 (2)	0.041 (3)	0.000 (2)	0.002 (2)	0.012 (2)
C431	0.041 (3)	0.028 (2)	0.033 (3)	−0.002 (2)	0.002 (2)	0.010 (2)

### Geometric parameters (Å, °)

**Table:**

S1—C11	1.782 (4)	S3—C31	1.773 (5)
S1—S2	2.0218 (16)	S3—S4	2.0133 (16)
S2—C21	1.766 (5)	S4—C41	1.770 (4)
N1—O14	1.204 (5)	N3—O33	1.205 (4)
N1—O13	1.211 (5)	N3—O34	1.235 (4)
N1—C14	1.467 (5)	N3—C34	1.461 (5)
N2—O23	1.211 (4)	N4—O43	1.231 (4)
N2—O24	1.233 (4)	N4—O44	1.235 (5)
N2—C24	1.457 (5)	N4—C44	1.433 (5)
O11—C131	1.274 (5)	O31—C331	1.302 (5)
O11—H1A	0.8200	O31—H3A	0.8200
O12—C131	1.256 (5)	O32—C331	1.238 (5)
O21—C231	1.263 (5)	O41—C431	1.303 (5)
O21—H2A	0.8200	O41—H4A	0.8200
O22—C231	1.245 (5)	O42—C431	1.216 (5)
C11—C16	1.387 (6)	C31—C36	1.388 (6)
C11—C12	1.391 (5)	C31—C32	1.393 (5)
C12—C13	1.390 (5)	C32—C33	1.393 (6)
C12—H12	0.9300	C32—H32	0.9300
C13—C14	1.386 (6)	C33—C34	1.400 (6)
C13—C131	1.489 (6)	C33—C331	1.480 (5)
C14—C15	1.379 (5)	C34—C35	1.359 (5)
C15—C16	1.374 (5)	C35—C36	1.388 (6)
C15—H15	0.9300	C35—H35	0.9300
C16—H16	0.9300	C36—H36	0.9300
C21—C22	1.400 (5)	C41—C42	1.385 (5)
C21—C26	1.409 (6)	C41—C46	1.394 (6)
C22—C23	1.386 (6)	C42—C43	1.374 (6)
C22—H22	0.9300	C42—H42	0.9300
C23—C24	1.386 (6)	C43—C44	1.403 (5)
C23—C231	1.496 (5)	C43—C431	1.498 (5)
C24—C25	1.378 (5)	C44—C45	1.388 (6)
C25—C26	1.366 (6)	C45—C46	1.390 (6)
C25—H25	0.9300	C45—H45	0.9300
C26—H26	0.9300	C46—H46	0.9300
			
C11—S1—S2	106.97 (15)	C31—S3—S4	105.94 (15)
C21—S2—S1	105.54 (15)	C41—S4—S3	106.38 (14)
O14—N1—O13	123.9 (4)	O33—N3—O34	123.4 (4)
O14—N1—C14	119.1 (4)	O33—N3—C34	119.0 (4)
O13—N1—C14	116.9 (4)	O34—N3—C34	117.5 (4)
O23—N2—O24	123.8 (4)	O43—N4—O44	122.6 (4)
O23—N2—C24	118.8 (4)	O43—N4—C44	118.9 (4)
O24—N2—C24	117.4 (4)	O44—N4—C44	118.5 (4)
C131—O11—H1A	109.5	C331—O31—H3A	109.5
C231—O21—H2A	109.5	C431—O41—H4A	109.5
C16—C11—C12	120.1 (4)	C36—C31—C32	120.0 (4)
C16—C11—S1	124.8 (3)	C36—C31—S3	125.5 (3)
C12—C11—S1	115.1 (3)	C32—C31—S3	114.6 (4)
C13—C12—C11	120.8 (4)	C31—C32—C33	120.1 (4)
C13—C12—H12	119.6	C31—C32—H32	119.9
C11—C12—H12	119.6	C33—C32—H32	119.9
C14—C13—C12	117.9 (4)	C32—C33—C34	118.2 (4)
C14—C13—C131	124.9 (4)	C32—C33—C331	119.6 (4)
C12—C13—C131	117.1 (4)	C34—C33—C331	122.3 (4)
C15—C14—C13	121.3 (4)	C35—C34—C33	122.1 (4)
C15—C14—N1	116.8 (4)	C35—C34—N3	117.4 (4)
C13—C14—N1	121.8 (4)	C33—C34—N3	120.4 (4)
C16—C15—C14	120.5 (4)	C34—C35—C36	119.4 (4)
C16—C15—H15	119.8	C34—C35—H35	120.3
C14—C15—H15	119.8	C36—C35—H35	120.3
C15—C16—C11	119.3 (4)	C35—C36—C31	120.2 (4)
C15—C16—H16	120.4	C35—C36—H36	119.9
C11—C16—H16	120.4	C31—C36—H36	119.9
C22—C21—C26	118.9 (4)	C42—C41—C46	120.1 (4)
C22—C21—S2	116.9 (3)	C42—C41—S4	115.9 (3)
C26—C21—S2	124.1 (3)	C46—C41—S4	124.0 (3)
C23—C22—C21	120.8 (4)	C43—C42—C41	121.5 (4)
C23—C22—H22	119.6	C43—C42—H42	119.2
C21—C22—H22	119.6	C41—C42—H42	119.2
C24—C23—C22	118.0 (4)	C42—C43—C44	117.8 (4)
C24—C23—C231	122.4 (4)	C42—C43—C431	118.4 (3)
C22—C23—C231	119.1 (4)	C44—C43—C431	123.7 (4)
C25—C24—C23	122.5 (4)	C45—C44—C43	121.9 (4)
C25—C24—N2	117.7 (4)	C45—C44—N4	118.3 (4)
C23—C24—N2	119.7 (4)	C43—C44—N4	119.6 (4)
C26—C25—C24	119.2 (4)	C44—C45—C46	119.0 (4)
C26—C25—H25	120.4	C44—C45—H45	120.5
C24—C25—H25	120.4	C46—C45—H45	120.5
C25—C26—C21	120.6 (4)	C45—C46—C41	119.7 (4)
C25—C26—H26	119.7	C45—C46—H46	120.2
C21—C26—H26	119.7	C41—C46—H46	120.2
O12—C131—O11	124.3 (4)	O32—C331—O31	124.1 (4)
O12—C131—C13	119.0 (3)	O32—C331—C33	121.2 (5)
O11—C131—C13	116.6 (4)	O31—C331—C33	114.6 (4)
O22—C231—O21	125.0 (4)	O42—C431—O41	125.6 (4)
O22—C231—C23	117.6 (3)	O42—C431—C43	121.3 (4)
O21—C231—C23	117.2 (4)	O41—C431—C43	113.0 (4)

### Hydrogen-bond geometry (Å, °)

**Table:**

*D*—H···*A*	*D*—H	H···*A*	*D*···*A*	*D*—H···*A*
O11—H1A···O22^i^	0.82	1.83	2.635 (4)	166
O21—H2A···O12^ii^	0.82	1.88	2.688 (4)	169
O31—H3A···O42^i^	0.82	1.85	2.666 (4)	171
O41—H4A···O32^ii^	0.82	1.83	2.618 (4)	160

Symmetry codes: (i) *x*, *y*+1, *z*; (ii) *x*, *y*−1, *z*.

## References

[bb1] Bruker (2004). *APEX2* and *SAINT* Bruker AXS Inc., Madison, Wisconsin, USA.

[bb2] Burnett, M. N. & Johnson, C. K. (1996). *ORTEPIII* Report ORNL-6895. Oak Ridge National Laboratory, Tennessee, USA.

[bb3] Farrugia, L. J. (1997). *J. Appl. Cryst.* **30**, 565.

[bb4] Gudbjarlson, H., Poirier, K. M. & Zaworotko, M. J. (1991). *J. Am. Chem. Soc.* **121**, 2599–2600.

[bb5] Li, H., Zhu, G. S., Guo, X. D., Sun, F. X., Ren, H. & Qiu, S. L. (2006). *Eur. J. Inorg. Chem.* pp. 4123–4128.

[bb6] Luo, F., Zheng, J. M. & Batten, S. R. (2007). *Chem. Commun* pp. 3744–3746. 10.1039/b706177c 17851614

[bb7] Shefter, E. & Kalman, T. I. (1969). *J. Chem. Soc. D*, p. 1027.

[bb8] Sheldrick, G. M. (1996). *SADABS* University of Göttingen, Germany.

[bb9] Sheldrick, G. M. (2008). *Acta Cryst.* A**64**, 112–122. 10.1107/S0108767307043930 18156677

[bb10] Spek, A. L. (2003). *J. Appl. Cryst.* **36**, 7–13.

[bb11] Ye, B. H., Tong, M. L. & Chen, X. M. (2005). *Coord. Chem. Rev.* **249**, 545–565.

